# The Axon-Protective WLD^S^ Protein Partially Rescues Mitochondrial Respiration and Glycolysis After Axonal Injury

**DOI:** 10.1007/s12031-014-0440-2

**Published:** 2014-10-29

**Authors:** Katharina Godzik, Michael P. Coleman

**Affiliations:** The Babraham Institute, Babraham Research Campus, Cambridge, CB22 3AT UK

**Keywords:** Axon injury, WLD^S^, ATP, Bioenergetics

## Abstract

The axon-protective Wallerian degeneration slow (WLD^S^) protein can ameliorate the decline in axonal ATP levels after neurite transection. Here, we tested the hypothesis that this effect is associated with maintenance of mitochondrial respiration and/or glycolysis. We used isolated neurites of superior cervical ganglion (SCG) cultures in the Seahorse XF-24 Metabolic Flux Analyser to determine mitochondrial respiration and glycolysis under different conditions. We observed that both mitochondrial respiration and glycolysis declined significantly during the latent phase of Wallerian degeneration. WLD^S^ partially reduced the decline both in glycolysis and in mitochondrial respiration. In addition, we found that depleting NAD levels in uncut cultures led to changes in mitochondrial respiration and glycolysis similar to those rescued by WLD^S^ after cut, suggesting that the maintenance of NAD levels in *Wld* ^*S*^ neurites after axonal injury at least partially underlies the maintenance of ATP levels. However, by using another axon-protective mutation (*Sarm1* ^−/−^), we could demonstrate that rescue of basal ECAR (and hence probably glycolysis) rather than basal OCR (mitochondrial respiration) may be part of the protective phenotype to delay Wallerian degeneration. These findings open new routes to study glycolysis and the connection between NAD and ATP levels in axon degeneration, which may help to eventually develop therapeutic strategies to treat neurodegenerative diseases.

## Introduction

Axons depend on the supply of energy in form of ATP to fuel important processes such as axonal transport, maintenance of the resting membrane potential and neurotransmitter release. Failure to generate sufficient ATP results in irreversible axon damage (Shen and Goldberg 2012). Axon degeneration is also often associated with energy depletion caused by mitochondrial dysfunction (Court and Coleman 2012), reduction in substrate availability (Ebneter et al. 2011; Lee et al. 2012; Viader et al. 2011) and/or excessive energy consumption (Stys 2005). In many neurological disorders, axon degeneration is an early event preceding neuronal cell body loss (Coleman and Freeman 2010). Understanding the mechanisms that underlie axon degeneration is therefore an important step towards developing novel therapeutic strategies for these diseases.

One important tool to study molecular mechanisms underlying axon degeneration is the Wallerian degeneration slow (*Wld* ^*S*^) mutant mouse, in which axon degeneration after injury is significantly delayed (Lunn et al. 1989) and in which axons are protected against degeneration in a variety of experimental models of neurological disorders such as models for multiple sclerosis (Kaneko et al. 2006), Parkinson’s disease (Sajadi et al. 2004; Hasbani and O’Malley 2006; Antenor-Dorsey and O’Malley 2012) and glaucoma (Howell et al. 2007). The *Wld* ^*S*^ phenotype is caused by expression of the chimeric WLD^S^ protein, which consists of the 70 amino acids of the N-terminal region of the ubiquitin ligase E4b (Ube4b) linked via 18 amino acids to full-length nicotinamide mononucleotide adenylyltransferase 1 (NMNAT1) (Coleman et al. 1998). Both parts of the WLD^S^ protein are important for the axon-protective phenotype (Coleman and Freeman 2010). WLD^S^ is thought to act in the axon through its NMNAT activity, converting nicotinamide mononucleotide (NMN) and ATP to NAD and PPi.

It has been shown that WLD^S^ can ameliorate the decline in NAD and ATP levels after axonal injury in primary culture (Wang et al. 2005), but the exact mechanisms through which ATP maintenance fails are not yet fully understood. Therefore, the aim of the present study was to investigate cellular energetics after axonal injury and the influence of WLD^S^ expression and NAD on these processes.

## Material and Methods

### Animals

All animal work was carried out in accordance with the UK Animals (Scientific Procedures) Act, 1986, under Project Licences 80/2254 and 70/7620 and was approved by the Babraham Institute Animal Welfare, Experimentation and Ethics Committee. C57BL/6JBabr and homozygous C57BL/6OlaHsd-Wld (*Wld* ^*S*^) mice were originally obtained from Harlan UK (Bicester, UK) and maintained as a long-term breeding colony at the Babraham Institute. *Sarm1* ^−/−^ mice (Kim et al. 2007) were obtained from Professor Marc Freeman (University of Massachusetts Medical School, Worcester, MA, USA) with permission from Professor Aihao Ding (Weill Cornell Medical College, New York, NY, USA).

### Cell Culture

Superior cervical ganglia (SCGs) were dissected from P0-P3 mouse pups. In each experiment, three cleaned explants were placed in the centre of each well in a 24-well plate (Seahorse Bioscience, North Billerica, MA, USA) pre-coated with poly-L-lysine (20 μg/ml for 1–2 h; Sigma) and laminin (20 μg/ml for 1–2 h; Sigma) to ensure the same amount of material in each well, and similar degrees of neurite outgrowth were confirmed for each genotype. Explants were cultured in Dulbecco’s modified Eagle’s medium (DMEM) with 4,500 mg/l glucose and 110 mg/l sodium pyruvate (Sigma), 2 mM glutamine, 1 % penicillin/streptomycin, 100 ng/ml 7S NGF (all Invitrogen), and 10 % foetal bovine serum (Sigma) at 37 °C and 5 % CO_2_. To reduce proliferation and viability of small numbers of non-neuronal cells, 4 μM aphidicolin (Calbiochem) was added. Cultures were used after 6 days in vitro.

### HPLC Analysis

NAD levels were determined using reversed phase HPLC as described before (Balducci et al. 1995). Briefly, SCG explant cultures treated for 24 h with DMSO or 100 nM FK866 were lysed, acidified with HClO_4_ and then neutralised with K_2_CO_3_ before loading on a LC18 Supelcosil column.

### Seahorse XF-24 Metabolic Flux Analysis

On the day of analysis, the medium of the cells was changed to unbuffered DMEM (modified DMEM supplemented with 25 mM glucose and 1 mM sodium pyruvate (both Sigma), pH 7.4). SCG explants were then incubated in a non-CO_2_ incubator at 37 °C for 30 min before cutting the neurites and removing the cell body mass. Remaining neurites were either measured straight after cut (early after cut) or left for another 140 min in the incubator before measuring (late after cut). As transected neurites degenerate after more than 4 h (Gilley and Coleman 2010), this ensures that the 80-min measurement period is complete during the time when distal neurites remain continuous. In some cases, neurites and cell bodies were not separated but treated with 100 nM of the specific NAMPT inhibitor FK866 (kind gift from RTI International, Research Park, NC, USA) 24 h prior to measurements. After a 20-min calibration step in the Seahorse XF-24 analyser, the analysis was started with a pattern of 2-min mixing, 2-min waiting, and 2-min measuring. Three baseline measurements of oxygen consumption rate (OCR) and extracellular acidification rate (ECAR) were taken before sequential injection of mitochondrial inhibitors. After each addition of mitochondrial inhibitor, three readings were taken before injection of the subsequent inhibitor. The mitochondrial inhibitors were used in the following order: oligomycin (2 μM), FCCP (8 μM), and rotenone (200 nM) together with antimycin (8 μM) (all from Sigma). OCR and ECAR were automatically calculated and recorded by the Seahorse XF-24 software. As some proteins are rapidly degraded following neurite transection (Gilley and Coleman 2010; Shen et al. 2013), readings were not normalised to protein levels, but rather, triplicate samples were run in each of up to six experiments and similar growth of neurites was confirmed.

### Statistical Analysis

All OCR and ECAR graphs were analysed using a two-way repeated measures ANOVA considering the different time and treatment/genotype as factors and matching samples for time point and day of experiment. This meant that day-to-day experimental variation was taken into account. However, for the sake of clarity, this matching was not reflected in the graphs, which simply display mean ± SEM for data collected on different days.

## Results

ATP levels significantly decline after axonal injury in primary culture neurites (Wang et al. 2005), but whether mitochondrial respiration, glycolysis or both are primarily affected is unknown. To investigate these processes, we used the Seahorse XF-24 Metabolic Flux Analyser to determine changes in oxygen consumption (parameter for mitochondrial respiration) and free protons (parameter for glycolysis) after axonal injury but before degeneration occurred. In addition to basal respiration and basal glycolysis, this technique enables the measurements of further parameters of mitochondrial respiration and glycolysis through serial injection of mitochondrial inhibitors such as oligomycin, FCCP, rotenone and antimycin. Oligomycin inhibits the ATP synthase allowing the determination of the amount of oxygen used to produce ATP under normal conditions. To compensate, glycolysis is upregulated, allowing measurement of maximal production of free protons. FCCP, an uncoupling agent, abolishes the proton gradient across the mitochondrial inner membrane leading to the maximum consumption of oxygen by the mitochondria as the electron transport system attempts to restore the gradient. Complete inhibition of mitochondrial respiration is achieved by blocking complex I (rotenone) and complex III (antimycin) simultaneously. Under this condition, the level of non-mitochondrial respiration can be determined.

**Fig. 1:**
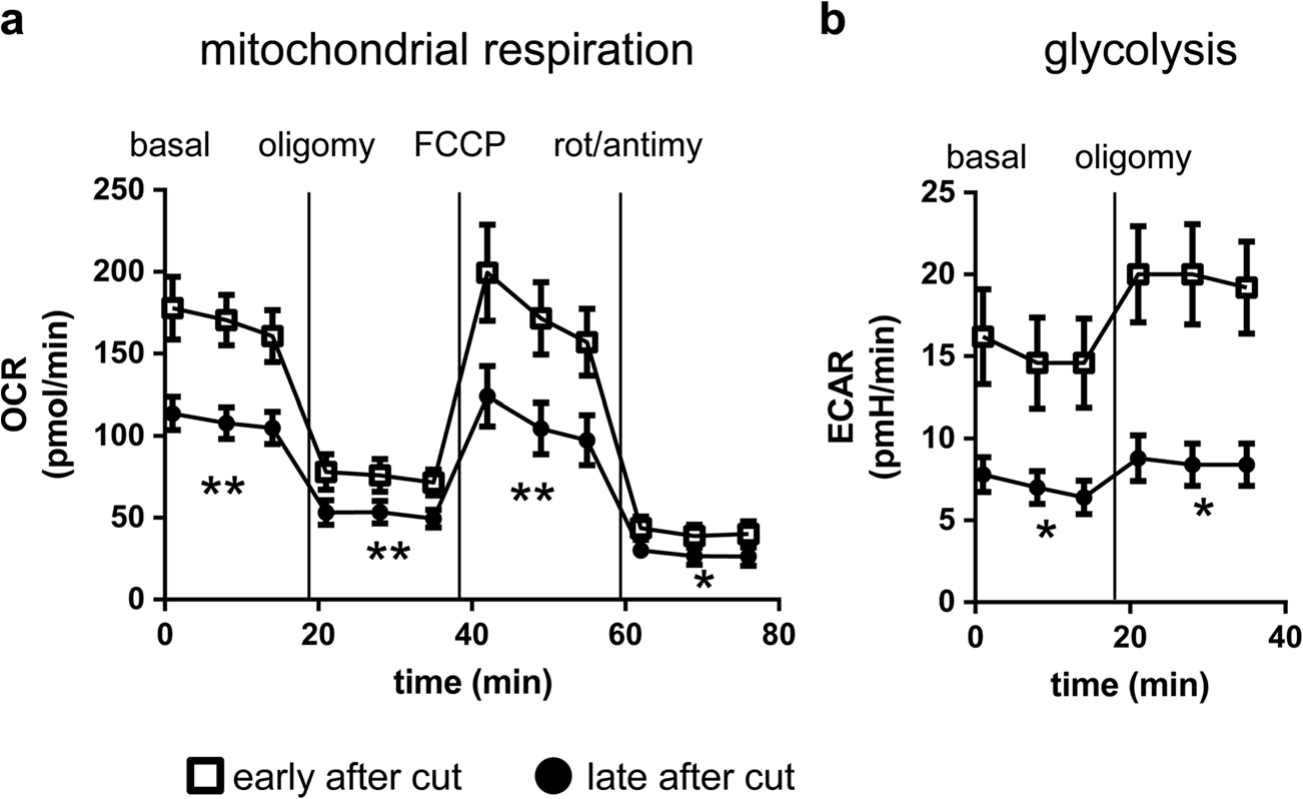
Mitochondrial respiration and glycolysis both decline after axonal injury. Oxygen consumption rate (OCR) in pmol/min (**a**) and extracellular acidification rate (ECAR) in pmH/min (**b**) of 6 days in vitro (DIV) mouse wild-type neurites measured early after cut (*square*) or late after cut (*circle*). Recordings took place under basal, oligomycin-inhibited (oligomy), FCCP-induced maximal and rotenone + antimycin A-inhibited (rot/antimy) conditions. *p < 0.05, **p < 0.01 two-way repeated measures ANOVA. Data are mean ± SEM of *n* = 5 independent experiments for both OCR and ECAR

Neurites of SCG explant cultures were subjected to these treatments at two time points: straight after cut (early) and 140 min after cut (late). Neurites that were measured late after cut showed significantly reduced mitochondrial respiration (Fig. 1a) and glycolysis (Fig. 1b) under all conditions of the assay compared to neurites that were measured early after cut. This finding suggests that reductions in both, mitochondrial respiration and glycolysis, contribute to the decline in ATP seen after axonal injury.

**Fig. 2:**
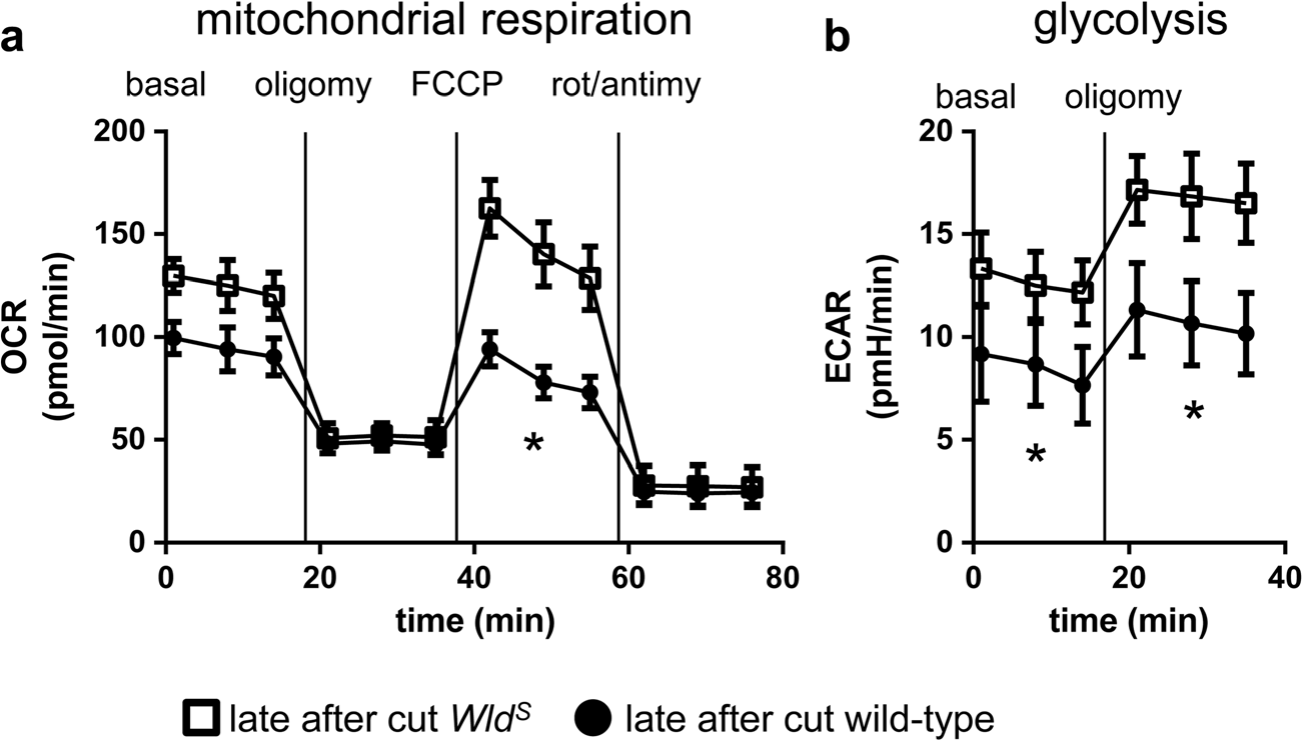
WLD^S^ partially rescues cellular energetics after axonal injury. OCRs in pmol/min (**a**) and ECARs in pmH/min (**b**) of six DIV mouse wild-type neurites (*circle*) and *Wld* ^*S*^ neurites (*square*) measured late after cut. Recordings took place under basal, oligomycin-inhibited (oligomy), FCCP-induced maximal and rotenone + antimycin A-inhibited (rot/antimy) conditions. *p < 0.05 two-way repeated measures ANOVA. Data are mean ± SEM of *n* = 6 independent experiments for both OCR and ECAR

As WLD^S^ expression can ameliorate the decline of ATP levels after axonal injury, we asked whether this is achieved by maintaining mitochondrial respiration and/or glycolysis. For this purpose, wild-type and *Wld* ^*S*^ neurites of SCG cultures were measured late after cut with the same series of treatments as described above. *Wld* ^*S*^ neurites showed significantly more oxygen consumption compared to wild-type neurites under maximal respiration condition. Under basal conditions, a non-significant trend towards increased oxygen consumption was also observed (Fig. 2a). Extracellular acidification rates were significantly increased under conditions of both basal and maximal glycolysis in cut *Wld* ^*S*^ neurites (Fig. 2b). These findings suggest that WLD^S^ slows the decline in ATP after cut by partially maintaining both glycolysis and mitochondrial respiration, although a block with 2-deoxyglucose would be required to rule out other causes of the increase in ECAR. No intrinsic differences in mitochondrial respiration or glycolysis were observed between *Wld* ^*S*^ and wild-type uncut SCG cultures (data not shown).

**Fig. 3:**
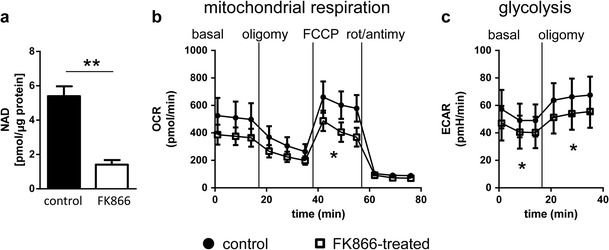
A reduction in NAD levels decreases cellular energetics. SCG explants were cultured for five DIV and treated with DMSO or 100 nM FK866 for 24 h as indicated. **a** NAD levels normalised to total protein (*n* = 3 per condition). ** *p* < 0.01 (Student’s *t* test). OCRs in pmol/min (**b**) and ECARs in pmH/min (**c**) of six DIV mouse wild-type SCG explants treated with DMSO (*circle*) or with 100 nM FK866 for 24 h (*square*). Recordings took place under basal, oligomycin-inhibited (oligomy), FCCP-induced maximal and rotenone + antimycin A-inhibited (rot/antimy) conditions. *p < 0.05 two-way repeated measures ANOVA. Data are mean ± SEM of *n* = 4 independent experiments for both OCR and ECAR. Higher OCR and ECAR readings were recorded as cell bodies were still present in these cultures

*Wld* ^*S*^ neurites can also maintain NAD levels after injury in vitro (Wang et al. 2005), as can transected nerves in vivo (Di Stefano et al. 2014). As both glycolysis and mitochondrial respiration require NAD, we tested whether these changes in axonal energetics could be explained by changes in NAD levels. For this, we decreased NAD levels in uncut SCG explant cultures by adding FK866, an inhibitor of NAMPT, the rate-limiting enzyme in the NAD salvage pathway, and subjected them to the assay described above. As expected, treatment with 100 nM FK866 for 24 h significantly decreased NAD levels in uncut SCG explants (Fig. 3a), confirming efficacy of the drug. Uncut SCG cultures treated with 100 nM FK866 remained viable for at least 3 days in culture (Di Stefano et. al 2014 and data not shown). Oxygen consumption under maximal respiration conditions was significantly reduced in uncut FK866-treated SCG neuron cultures compared to control cultures (Fig. 3b). Extracellular acidification rates under conditions of basal and maximal glycolysis were also significantly reduced in FK866-treated cultures (Fig. 3c). Thus, lowering NAD levels is sufficient to produce changes in mitochondrial respiration and glycolysis similar to those rescued by WLD^S^ after cut.

**Fig. 4:**
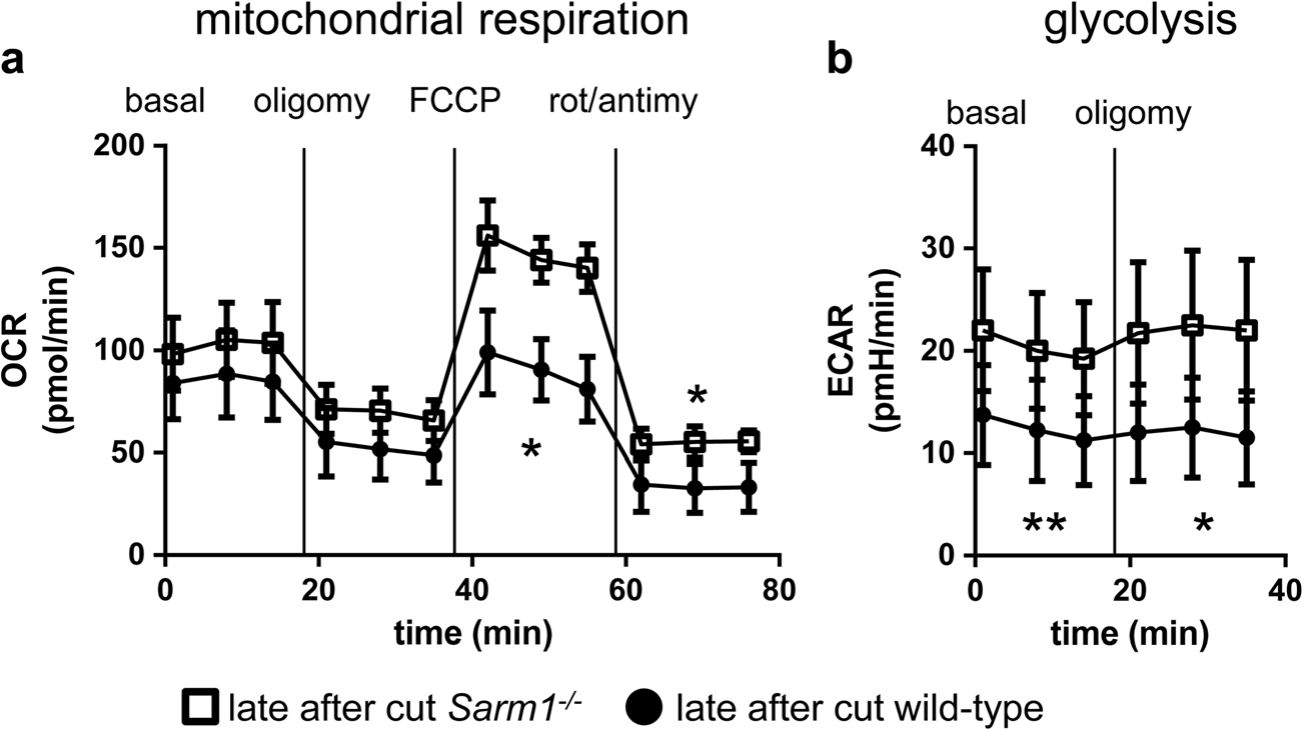
Rescue of basal glycolysis rather than basal mitochondrial respiration may be part of the protective phenotype to delay Wallerian degeneration. OCRs in pmol/min (**a**) and ECARs in pmH/min (**b**) of six DIV mouse wild-type neurites (*circle*) and *Sarm1* ^−/−^ neurites (*square*) measured late after cut. Recordings took place under basal, oligomycin-inhibited (oligomy), FCCP-induced maximal and rotenone + antimycin A-inhibited (rot/antimy) conditions. *p < 0.05, **p < 0.01 two-way repeated measures ANOVA. Data are mean ± SEM of *n* = 4 independent experiments for both OCR and ECAR

One question that remains is whether the partial rescue of mitochondrial respiration and glycolysis in WLD^S^ neurites underlies the protective phenotype of WLD^S^. To further investigate this matter, we used another genetic model to delay Wallerian degeneration (*Sarm1* ^−/−^) (Osterloh et al. 2012). SARM1 is a Toll-like receptor adaptor protein that plays a role in the innate immune system (Dalod 2007). Recently, it was discovered that *Sarm1* deletion can significantly delay Wallerian degeneration similar to WLD^S^ (Osterloh et al. 2012; Gerdts et al. 2013). To investigate whether mitochondrial respiration and glycolysis are also partially rescued in *Sarm1* ^−/−^ neurites, we subjected cut *Sarm1* ^−/−^ neurites to the same assay described above. *Sarm1* ^−/−^ neurites showed no change in oxygen consumption under basal conditions compared to wild-type neurites (Fig. 4a) suggesting that rescue of basal mitochondrial respiration is not necessary to delay Wallerian degeneration. Interestingly, *Sarm1* ^−/−^ neurites showed an increased maximal mitochondrial respiration similar to the one seen in *Wld* ^*S*^ neurites. Extracellular acidification rates were increased relative to wild-type under both basal and maximal conditions (Fig. 4b) which was again similar to the observation in *Wld* ^*S*^ neurites. This indicates that amelioration of defects in glycolysis may play a role in the protective phenotype to delay Wallerian degeneration.

## Discussion

This study reports that both mitochondrial respiration and glycolysis are reduced in neurites after axonal injury, a finding that could help explain the decline in ATP levels after axonal injury observed previously (Wang et al. 2005). Thus, a decline in both mitochondrial respiration and glycolysis appears to contribute to the reduction in ATP. Although the experiments reported in Wang et al. (2005) were based on DRG cultures and we used SCGs, many studies have shown that the *Wld* ^*S*^ (and *Sarm1* ^−/−^) phenotype is present in both cell culture systems (Gilley et al. 2013; Sasaki and Milbrandt 2010; Babetto et al. 2010; Milde et al. 2013; Gilley and Coleman 2010; Conforti et al. 2011; Osterloh et al. 2012). WLD^S^ also preserves ATP levels after energy deprivation in cortical neuron cultures (Shen et al. 2013) and extends neurite survival in dopaminergic neurons exposed to the mitochondrial toxin MPP^+^ (Antenor-Dorsey and O’Malley 2012). Thus, it is likely that these data are relevant to many neuronal cell types throughout the central, peripheral and sympathetic nervous system.

In neurites expressing WLD^S^, the decline in mitochondrial respiration and glycolysis was partially ameliorated. This observation is in line with studies showing that WLD^S^-expressing neurites can maintain ATP levels after axonal injury (Wang et al. 2005). Our results indicate that glycolysis in particular, and potentially also mitochondrial respiration, contribute to this effect. However, another study suggested that WLD^S^ expression is not able to slow the rate of a rotenone-induced reduction in neuronal ATP (Press and Milbrandt 2008). These contradictory findings may be explained by the different trigger of axon degeneration in these two studies (i.e. mitochondrial damage vs axotomy).

We were able to demonstrate that a reduction in NAD levels is sufficient to reduce cellular energetics. Interestingly, these changes were similar to those rescued by WLD^S^ after cut. This suggests that the maintenance of NAD levels in *Wld* ^*S*^ neurites after axonal injury at least partially contributes to the maintenance of ATP levels.

NAD is an important redox equivalent in glycolysis as well as in mitochondrial respiration. Thus, a depletion of cytosolic NAD caused by FK866 or axonal injury would directly influence glycolysis. However, the mitochondrial NAD pool is thought to be independent of the cytosol due to the impermeability of the mitochondrial inner membrane to NAD (Nikiforov et al. 2011; Di Lisa and Ziegler 2001). NAD is thought to be synthesised in the mitochondrial matrix by the mitochondrial specific isoform NMNAT3, using NMN that enters mitochondria by an unknown mechanism. The existence of such an isoform has recently been challenged questioning the local synthesis of NAD in mitochondria (Felici et al. 2013). Further work aimed at understanding compartmentalisation and flux of NAD in the cell should provide valuable insight to test the hypothesis that low NAD levels contribute to the decline in ATP levels after axonal injury.

However, the contention that maintenance of NAD levels is critical for prolonged axon survival in WLD^S^-expressing neurons is controversial. Exogenous application of NAD (Araki et al. 2004; Wang et al. 2005; Sasaki et al. 2006) and its precursors such as Nam (Wang et al. 2005), NaMN (Sasaki et al. 2006), NMN (Sasaki et al. 2006) and NR (Sasaki et al. 2006) led to axonal protection in vitro. In a mouse model of EAE, application of Nam prevented the degeneration of demyelinated axons and improved behavioural deficits (Kaneko et al. 2006). Overexpression of enzymes upstream of NMNAT2 in the NAD biosynthesis pathway, such as nicotinic acid phosphoribosyltransferase and NAMPT, also showed a moderate protective effect in vitro (Sasaki et al. 2006). However, no axon-protective effect was observed when NAD levels were increased by inhibiting the NAD hydrolysing enzymes PARP and/or CD38 (Sasaki et al. 2009). It was argued that these contradictory findings reflect changes in NAD levels in different subcellular compartments (Wang et al. 2012). Finally, in a recent study, we show that a rise in the NMNAT substrate NMN is more closely linked to axon degeneration than a fall in NAD (DiStefano et al. 2014).

With this study, we also tried to address whether partial rescue of mitochondrial respiration and glycolysis is a mechanism by which WLD^S^ promotes axon survival. Our data in another genetic model of delayed Wallerian degeneration (*Sarm1* ^−/−^) indicated that ameliorating the defects in basal mitochondrial respiration is not necessary to delay Wallerian degeneration questioning the importance of ATP generated through mitochondrial respiration for axon survival. This is in line with a study in flies that mislocalised axonal mitochondria to the cell body. In this scenario, WLD^S^ was still able to delay Wallerian degeneration after axonal injury (Kitay et al. 2013) suggesting that the protective phenotype is independent of mitochondria. In our study, we could also demonstrate that the decline in glycolysis under basal and maximal conditions was ameliorated in *Wld* ^*S*^ and *Sarm1* ^−/−^ neurites. Thus, ATP production through glycolysis could be an underlying mechanism promoting axonal survival, but we cannot rule out the possibility that it is a consequence of survival. A previous study showed that a reduction in ATP levels by inhibition of glycolysis does not cause axon degeneration (Press and Milbrandt 2008) questioning the importance of ATP generated by glycolysis for axons. A recent study suggested that neither mitochondrial respiration nor glycolysis is important to maintain ATP levels after energy deprivation. Instead, the reverse action of enzymes such as NMNATs was considered to be important for ATP production after energy deprivation (Shen et al. 2013).

In this study, we were able to show that cellular energetics decline within the first 140 min after axonal injury in primary culture and that WLD^S^ can partially rescue this decline, potentially through maintaining NAD levels. However, it is not necessary to rescue basal mitochondrial respiration to prolong the survival of injured axons. Glycolysis, on the other hand, appears more closely related to survival of these axons. These findings open new routes to study glycolysis and the connection between NAD and ATP levels in axon degeneration, which may help to eventually develop therapeutic strategies to treat neurodegenerative diseases.

## Abbreviations

DIV: Days in vitro
ECAR: Extracellular acidification rate
NAMPT: Nicotinamide phosphoribosyltransferase
NMN: Nicotinamide mononucleotide
NMNAT: Nicotinamide adenylyltransferase
OCR: Oxygen consumption rate
Ube4b: Ubiquitin ligase E4b Superior cervical ganglion
SCG: Superior cervical ganglion
WLD^S^: Wallerian degeneration slow protein

## References

[CR1] Antenor-Dorsey JA, O’Malley KL (2012). WldS but not Nmnat1 protects dopaminergic neurites from MPP + neurotoxicity. Mol Neurodegener.

[CR2] Araki T, Sasaki Y, Milbrandt J (2004). Increased nuclear NAD biosynthesis and SIRT1 activation prevent axonal degeneration. Science.

[CR3] Babetto E, Beirowski B, Janeckova L (2010). Targeting NMNAT1 to axons and synapses transforms its neuroprotective potency in vivo. J Neurosci.

[CR4] Balducci E, Orsomando G, Polzonetti V (1995). NMN adenylyltransferase from bull testis: purification and properties. Biochem J.

[CR5] Coleman MP, Freeman MR (2010). Wallerian degeneration, wld(s), and nmnat. Annu Rev Neurosci.

[CR6] Coleman MP, Conforti L, Buckmaster EA (1998). An 85-kb tandem triplication in the slow Wallerian degeneration (Wlds) mouse. Proc Natl Acad Sci U S A.

[CR7] Conforti L, Janeckova L, Wagner D (2011). Reducing expression of NAD + synthesizing enzyme NMNAT1 does not affect the rate of Wallerian degeneration. FEBS J.

[CR8] Court FA, Coleman MP (2012). Mitochondria as a central sensor for axonal degenerative stimuli. Trends Neurosci.

[CR9] Dalod M (2007). Studies of SARM1 uncover similarities between immune and neuronal responses to danger. Sci STKE: Sig Transduct Knowl Environ.

[CR10] Di Lisa F, Ziegler M (2001). Pathophysiological relevance of mitochondria in NAD(+) metabolism. FEBS Lett.

[CR11] Di Stefano M, Nascimento-Ferreira I, Orsomando G et al (2014) A rise in NAD precursor nicotinamide mononucleotide (NMN) after injury promotes axon degeneration. Cell Death Differ (in press) 10.1038/cdd.2014.164 PMC4392071 25323584

[CR12] Ebneter A, Chidlow G, Wood JP, Casson RJ (2011). Protection of retinal ganglion cells and the optic nerve during short-term hyperglycemia in experimental glaucoma. Arch Ophthalmol.

[CR13] Felici R, Lapucci A, Ramazzotti M, Chiarugi A (2013). Insight into molecular and functional properties of NMNAT3 reveals new hints of NAD homeostasis within human mitochondria. PLoS One.

[CR14] Gerdts J, Summers DW, Sasaki Y, Diantonio A, Milbrandt J (2013). Sarm1-mediated axon degeneration requires both SAM and TIR interactions. J Neurosci.

[CR15] Gilley J., Coleman M. P. (2010) Endogenous Nmnat2 is an essential survival factor for maintenance of healthy axons. PLoS Biol 8 (1) doi:10.1371/journal.pbio.1000300 10.1371/journal.pbio.1000300 PMC2811159 20126265

[CR16] Gilley J, Adalbert R, Yu G, Coleman MP (2013). Rescue of peripheral and CNS axon defects in mice lacking NMNAT2. J Neurosci.

[CR17] Hasbani DM, O’Malley KL (2006). Wld(S) mice are protected against the Parkinsonian mimetic MPTP. Exp Neurol.

[CR18] Howell GR, Libby RT, Jakobs TC (2007). Axons of retinal ganglion cells are insulted in the optic nerve early in DBA/2 J glaucoma. J Cell Biol.

[CR19] Kaneko S, Wang J, Kaneko M (2006). Protecting axonal degeneration by increasing nicotinamide adenine dinucleotide levels in experimental autoimmune encephalomyelitis models. J Neurosci.

[CR20] Kim Y, Zhou P, Qian L (2007). MyD88-5 links mitochondria, microtubules, and JNK3 in neurons and regulates neuronal survival. J Exp Med.

[CR21] Kitay BM, McCormack R, Wang Y, Tsoulfas P, Zhai RG (2013). Mislocalization of neuronal mitochondria reveals regulation of Wallerian degeneration and NMNAT/WLD(S)-mediated axon protection independent of axonal mitochondria. Hum Mol Genet.

[CR22] Lee Y, Morrison BM, Li Y (2012). Oligodendroglia metabolically support axons and contribute to neurodegeneration. Nature.

[CR23] Lunn ER, Perry VH, Brown MC, Rosen H, Gordon S (1989). Absence of Wallerian degeneration does not hinder regeneration in peripheral-nerve. Eur J Neurosci.

[CR24] Milde S, Gilley J, Coleman MP (2013). Subcellular localization determines the stability and axon protective capacity of axon survival factor Nmnat2. PLoS Biol.

[CR25] Nikiforov A, Dolle C, Niere M, Ziegler M (2011). Pathways and subcellular compartmentation of NAD biosynthesis in human cells: from entry of extracellular precursors to mitochondrial NAD generation. J Biol Chem.

[CR26] Osterloh JM, Yang J, Rooney TM (2012). dSarm/Sarm1 is required for activation of an injury-induced axon death pathway. Science.

[CR27] Press C, Milbrandt J (2008). Nmnat delays axonal degeneration caused by mitochondrial and oxidative stress. J Neurosci.

[CR28] Sajadi A, Schneider BL, Aebischer P (2004). Wlds-mediated protection of dopaminergic fibers in an animal model of Parkinson disease. Curr Biol.

[CR29] Sasaki Y, Milbrandt J (2010). Axonal degeneration is blocked by nicotinamide mononucleotide adenylyltransferase (Nmnat) protein transduction into transected axons. J Biol Chem.

[CR30] Sasaki Y, Araki T, Milbrandt J (2006). Stimulation of nicotinamide adenine dinucleotide biosynthetic pathways delays axonal degeneration after axotomy. J Neurosci.

[CR31] Sasaki Y, Vohra BP, Lund FE, Milbrandt J (2009). Nicotinamide mononucleotide adenylyl transferase-mediated axonal protection requires enzymatic activity but not increased levels of neuronal nicotinamide adenine dinucleotide. J Neurosci.

[CR32] Shen H, Goldberg MP (2012). Creatine pretreatment protects cortical axons from energy depletion in vitro. Neurobiol Dis.

[CR33] Shen H, Hyrc KL, Goldberg MP (2013). Maintaining energy homeostasis is an essential component of Wld(S)-mediated axon protection. Neurobiol Dis.

[CR34] Stys PK (2005). General mechanisms of axonal damage and its prevention. J Neurol Sci.

[CR35] Viader A, Golden JP, Baloh RH, Schmidt RE, Hunter DA, Milbrandt J (2011). Schwann cell mitochondrial metabolism supports long-term axonal survival and peripheral nerve function. J Neurosci.

[CR36] Wang J, Zhai Q, Chen Y (2005). A local mechanism mediates NAD-dependent protection of axon degeneration. J Cell Biol.

[CR37] Wang JT, Medress ZA, Barres BA (2012). Axon degeneration: molecular mechanisms of a self-destruction pathway. J Cell Biol.

